# Long‐term effects of childhood trauma subtypes on adult brain function

**DOI:** 10.1002/brb3.2981

**Published:** 2023-03-27

**Authors:** Jianjian Cai, Jia Li, Dong Liu, Sikang Gao, Yunyun Zhao, Jiaxuan Zhang, Qin Liu

**Affiliations:** ^1^ Department of Radiology, Tongji Hospital, Tongji Medical College Huazhong University of Science and Technology Wuhan P. R. China

**Keywords:** abuse, childhood trauma, functional connectivity, graph analysis, neglect

## Abstract

**Introduction:**

Childhood trauma is prevalent in our society, whereas little is known about why and how different forms of early traumatic experiences exert long‐term effects on differential brain function in adulthood.

**Purpose:**

This study aimed to scale experience‐specific neural correlates of distinct subtypes of childhood trauma.

**Methods:**

We used resting‐state functional magnetic resonance imaging data from 216 adults with different degrees of childhood trauma. Graph analysis was combined with the Childhood Trauma Questionnaire to inspect the impact of distinct subtypes of childhood trauma on the brain.

**Results:**

We found that childhood trauma experiences have a detrimental effect on depression and anxiety behavior. On one hand, childhood neglect scores were positively associated with network transmission efficiency of regions involved in cognitive and executive functions, such as temporal lobe, insular cortex, and parahippocampal gyrus. On the other hand, childhood abuse scores were tightly linked to functional reorganizations of regions mediated by immature ego defense system and damaged emotion representation. Specifically, the abuse scores were positively associated with network transmission efficiency of the visual, auditory, linguistic, and motor cortex. Isolated communications in temporal cortex and supplementary motor cortex were related to emotional and physical abuse scores.

**Conclusions:**

Our data showed the differential associations of abusive and neglectful experiences with brain function in adulthood. These experience‐specific associations could underpin potentially differential risks of specific forms of psychiatric sequelae in adulthood. More attentions to maltreated children and timely psychological treatment are needed to reduce the incidence of psychosis.

## Graphical abstract

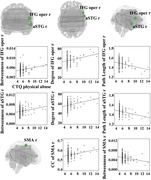

## 1 INTRODUCTION

Childhood trauma is associated with a predisposition to serious long‐term mental and physical ill‐health. More than 1 billion children and adolescents across the world are exposed to violent behavior (Hillis et al., 2016). Individuals who experience adverse conditions during childhood exhibit greater vulnerability for developing mental disorders later in adult (Misiak et al., 2017; Thumfart et al., 2022), such as PTSD (Klaming et al., 2019), anxiety (Ahmed‐Leitao et al., 2019), depressive disorders (Opel, Redlich, Dohm, et al., 2019), substance abuse (De Bellis et al., 2019), antisocial behavior (Busso et al., 2017), and personality disorders (Wells et al., 2020). The high prevalence of childhood trauma and its’ long‐lasting impact on both mental and physical health make it necessary to better understand the pathogenesis and development process, which is essential for early diagnostics, and for the development of interventions that can attenuate or overcome these pathologies. Therefore, the key to understand biological development process is to comprehensively explore the children‐trauma‐affected brain regions, facilitating better intervention strategies for those who have experienced trauma. Numerous neuroimaging studies indicated that childhood trauma could lead to detrimental alterations in brain regions, particularly in the amygdala, hippocampus, prefrontal cortex, insular, and cingulate gyrus, might imply that reward processing, emotional stimulation, cognitive regulation, and executive function are modulated (Cancel et al., 2019; Johnstone et al., 2016; Klaming et al., 2019; Opel, Redlich, Dohm, et al., 2019; Opel, Redlich, Repple, et al., 2019; Souza‐Queiroz et al., 2016; van Velzen et al., 2016). Diffusion tensor imaging studies had reported that interconnectivity of white matter fibers and isolated communication between regions were found in trauma‐experienced childhood (Eluvathingal et al., 2006; Jackowski et al., 2008; Monteleone et al., 2019; Tendolkar et al., 2018).

Generally, childhood trauma includes emotional abuse and neglect, physical abuse and neglect, and sexual abuse. Different forms of childhood trauma had distinct impacts on brain circuits (Blair et al., 2019; Dennison et al., 2019; Pollak et al., 2000; Sheridan et al., 2017). Different types of trauma together or only one or two forms of trauma used to create a risk score in previous studies (Busso et al., 2017; Cisler, 2017; Marusak et al., 2015; McCrory et al., 2013) failed to distinguish trauma‐type influence on brain (McLaughlin & Sheridan, 2016) as a critical factor for neural development in life. A more effective strategy to understand the pathogenesis and development process of psychiatric disorders is to identify neurobiological mechanism that is induced by a specific subtype of childhood trauma. A strong link between childhood trauma and increased risks of adult psychopathology drives many studies to investigate the associations of the childhood trauma with the onset and the severity of psychiatric disorders in adults. However, the existence and nature of biological alterations of childhood trauma beyond the impact on different psychiatric disorders are less clear. Functional magnetic resonance imaging (fMRI) is a prominent tool which helps in the noninvasive examination and localization of the intrinsic activity within the brain. Changes in regional function connectivity represent a layer of biological alterations that may link childhood trauma and brain functions. Graph theory is one of the most widely used methods for fMRI analysis. It reveals the complex connections between brain regions by establishing mathematical models of complex network functions (Smitha et al., 2017). In the current exploratory study utilizing graph analysis, we used resting‐state fMRI data from a large community sample to delineate distinct impacts of different subtypes of childhood trauma on brain network connectivity properties. We hypothesized that abusive forms and neglectful forms of childhood trauma would differ in their associations with atypical neural functioning.

## 2 MATERIALS AND METHODS

### 2.1 Participants

A total of 216 right‐handed healthy young adults were recruited in the study. All subjects completed a structured clinical interview with the Chinese version of the Mini International Neuropsychiatric Interview. Participants were carefully screened to ensure that they had no history of head trauma, genetic diseases, psychiatric treatment, psychiatric or neurological illness, drug, or alcohol abuse and had no contraindications to MRI examination. In order to avoid racial differences, only Han Chinese were included in this study. All subjects were strongly right‐handed according to the Chinese edition of the Edinburgh Handedness Inventory. The human experiment was approved by the Ethical Committee of Tongji Hospital of Tongji Medical College of Huazhong University of Science and Technology. All subjects gave written informed consent in accordance with the Declaration of Helsinki. All methods were carried out in accordance with approved institutional guidelines and regulations.

### 2.2 Questionnaires

In the present study, maltreatment experiences during childhood were assessed by administering the Chinese version of the Childhood Trauma Questionnaire (CTQ) that assesses childhood experiences of abuse or neglect in adults (Bernstein et al., 1997). The CTQ‐Short Form is a short version of the CTQ, which has been proved to be a standardized and fully effective tool for retrospective assessment of childhood adversity (Everaerd et al., 2016). The Beck Depression Inventory Second Edition (BDI‐II) was one of the most common measures of depressive symptoms in neurodegenerative diseases, which was a self‐assessment scale evaluating a relatively narrow range of symptoms also providing a rating of severity (Cuoco et al., 2021). The Spielberger State‐Trait Anxiety Inventory (STAI) was a well‐known 40‐item instrument, measuring respectively transient and enduring levels of anxiety (Kvaal et al., 2005). Participants also completed the BDI and STAI to further characterize subjects.

### 2.3 MRI and data preprocessing procedure

All scans were performed on a 3.0 Tesla MR system (Discovery MR750, General Electric, Milwaukee, WI, USA). Earplugs were used to reduce scanner noise, and tight yet comfortable foam paddings were used to minimize head motion. Resting‐state fMRI data were obtained using single‐shot echo‐planar imaging with the following parameters: repetition time (TR)/echo time (TE) = 2000/30 ms; field of view (FOV) = 220 mm × 220 mm; matrix = 64 × 64; slice thickness = 3 mm; no gap; flip angle (FA) = 90°, 36 interleaved transverse slices; 185 volumes. During the fMRI scans, all subjects were instructed to keep their body inactive, keep their eyes closed, think of nothing in particular, and not fall asleep. To better coregister the fMRI data, sagittal 3D T1‐weighted images were acquired using a brain volume sequence (TR/TE = 8.16/3.18 ms; inversion time = 450 ms; FA = 12 degree; FOV = 256 mm × 256 mm; matrix = 256 × 256; slice thickness = 1 mm; no gap; 188 sagittal slices).

The resting‐state fMRI data were preprocessed using SPM8 (http://www.fil.ion.ucl.ac.uk/spm). The first 10 volumes for each subject were discarded to allow the signal to reach equilibrium and the participants to adapt to the scanning noise. The remaining 175 volumes were then corrected for the acquisition time delay between slices. All fMRI data were within the defined motion thresholds (rotational or translational motion parameters lower than 2° or 2 mm). Framewise displacement indexes volume‐to‐volume changes in head position. These changes were obtained from the derivatives of the rigid body realignment estimates that were used to realign blood oxygen level‐dependent (BOLD) data during fMRI preprocessing (Power et al., 2012, 2013). The approach used to normalize these functional images included the following steps: (i) individual structural images were linear co‐registered to the mean functional image after motion correction; (ii) the transformed structural images were segmented into gray matter, white matter, and cerebrospinal fluid; and then gray matter was nonlinear co‐registered to the Montreal Neurological Institute (MNI) space; and (iii) the motion‐corrected functional volumes were spatially normalized to the MNI space using the parameters estimated during nonlinear co‐registration. The functional images were then resampled into a voxel size of 3 × 3 × 3 mm^3^. After normalization, images were smoothed using a Gaussian kernel of 8 × 8 × 8 mm^3^ full‐width at half‐maximum. The datasets were band‐pass filtered with frequency from 0.01 to 0.1 Hz, and several nuisance covariates (six motion parameters and average BOLD signals of the ventricular and white matter) were regressed out from the data.

### 2.4 Regions of interest (ROIs) definition and network construction

A total of 15 subcortical regions from FSL Harvard‐Oxford Atlas maximum likelihood subcortical atlas (HarvardOxford‐sub‐maxprob‐thr25‐1mm.nii) and 91 cortical regions from FSL Harvard‐Oxford Atlas maximum likelihood cortical atlas (HarvardOxford‐cort‐maxprob‐thr25‐1mm.nii) were defined as regions of interest (ROIs) (Whitfield‐Gabrieli & Nieto‐Castanon, 2012). We calculated the functional connectivity between each pair of the 106 ROIs. The functional connectivity was generated by averaging the BOLD time series separately in the two ROIs and then computing the Pearson's correlation coefficient between the two averaged time series. The resulting correlation was then transformed to approximate a Gaussian distribution using Fisher's *r*‐to‐*z* transformation. Thus, for each subject, we obtained a 106 × 106 matrix, with each element representing the connectivity strength between the corresponding two ROIs. For graph analysis, total 106 ROIs were defined as network nodes. We used the connectivity strength between any two ROI *i* and *j* to define the weights of network connections, building a weighted network. To remove the spurious connections, we used a minimum threshold (Huang et al., 2019) of functional connectivity (*w*_ij_ = 0.15, where *w*_ij_ was defined as the weight of the edge) between two nodes. Finally, for each subject, a weighted 106 × 106 functional network was constructed.

### 2.5 Statistical analysis

The following measures were calculated to characterize the topologic organization of the brain functional network: local efficiency, global efficiency, clustering coefficient, shortest path length, and small‐world parameters (Wang et al., 2010). The following measures were considered for regional nodal characteristics: betweenness centrality, clustering coefficient, shortest path length, and degree. To investigate whether the different CTQ subtypes were associated with values of network properties, multiple linear regression models were constructed in 216 subjects. Gender, education level, and age were also controlled in the multiple regression analyses. Statistical significance was set at *p* < .05 (FDR seed‐level correction). If significant effects were determined, linearly correlations between CTQ subtypes and network properties were presented in scatter diagrams to visually display the significant linear regression findings.

Statistical analyses for the demographic and cognitive data were performed using Statistical Package for the Social Sciences version 18.0 (SPSS, Chicago, IL, USA) for Windows. To further explore the relationship between depression/anxiety and childhood trauma, the partial correlation analysis were used to study correlations between BDI/STAI scores and CTQ scores. Gender, education level, and age were carried out as nuisance variables.

## 3 RESULTS

### 3.1 Demographic characteristics and behavioral results

Demographic and clinical characteristics of all children (*n* = 216) are shown in Table 1. For Pearson correlation analysis, CTQ‐sum scores were correlated with the STAI scores (*p* < .001), and four of the five CTQ subtypes were correlated positively with the STAI scores: emotional abuse, emotional neglect, physical neglect (all *p* < .001), and physical abuse (*p* < .01). In addition, the CTQ‐sum scores were correlated with the BDI score (*p* < .001), and four of the five CTQ subtypes were correlated positively with the BDI score: physical neglect (*p* < .01), sexual abuse (*p* < .05), emotional abuse and emotional neglect (both *p* < .001).

**TABLE 1:** Sociodemographic and clinical characteristics of the sample (*n* = 216).

Characteristics	Mean (SD)	Range	*r*	
			STAI scores	BDI score
Age (years)	24.1 (1.9)	20–30		
Gender (female)	158/216	–		
Education level (years)	17.7 (1.5)	13–22		
STAI scores	69.1 (14.8)	42–119		
BDI score	4.7 (4.9)	0–22		
CTQ total	30.4 (5.2)	25–49	.382^***^	.291^***^
Emotional abuse	6.2 (1.5)	5–11	.344^***^	.263^***^
Physical abuse	5.5 (1.3)	5–14	.180^**^	
Sexual abuse	5.2 (0.6)	5–9		.153^*^
Emotional neglect	7.7 (2.7)	5–17	.301^***^	.293^***^
Physical neglect	5.9 (1.4)	5–11	.245^***^	.187^**^

*Note*: ^***^ means *p* < .001, ^**^ means *p* < .01, ^*^ means *p* < .05.

### 3.2 Correlations between CTQ and network properties

Multiple linear regression models were used to assess the unique influences of CTQ scores on network properties. The sample showed a small‐world organization of functional network expressed by a *λ* ≈ 1 and *γ* > 1. There were no significant effects of CTQ subtypes on the network global efficiency, local efficiency, clustering coefficient, shortest path length, and small‐world parameters. For regional nodal characteristics, different CTQ subtypes were, respectively, associated with values of different network properties.

**FIGURE 1:**
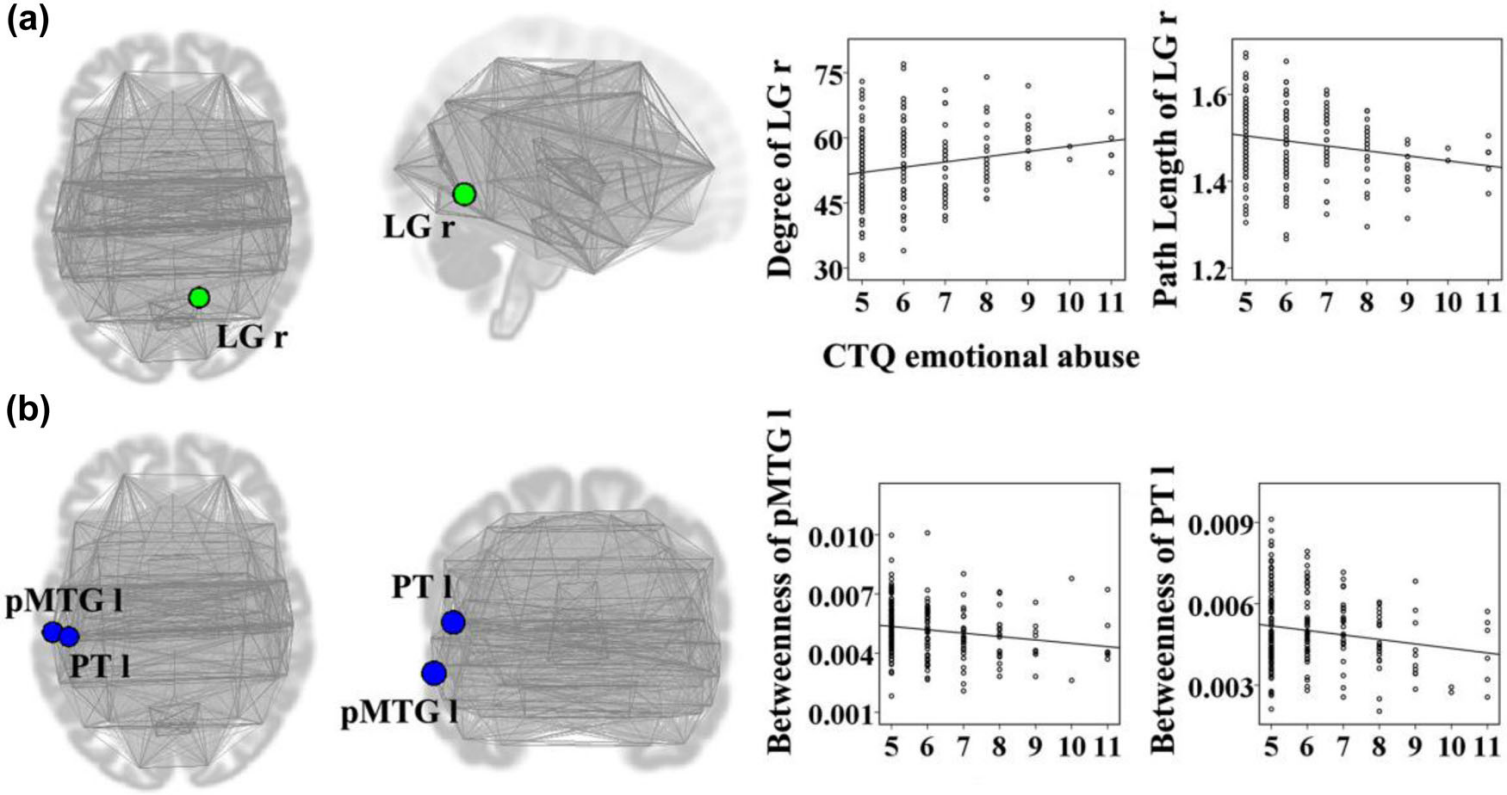
Correlations between the emotional abuse score and values of network properties. The scatter diagrams showed that the emotional abuse score was positively associated with degree of the LG r; path length of the LG r, betweenness of the pMTG l, and betweenness of the PT l were negatively correlated with the emotional abuse score. CTQ: Childhood Trauma Questionnaire; LG r: right lingual gyrus; pMTG l: left posterior middle temporal gyrus; PT l: left planum temporal. Part (a) shows the location of right LG as well as the correlations between its functional properties and the emotional abuse score. Part (b) shows the location of pMTG l and PT l as well as the correlations between their functional properties and the emotional abuse scores.

**FIGURE 2:**
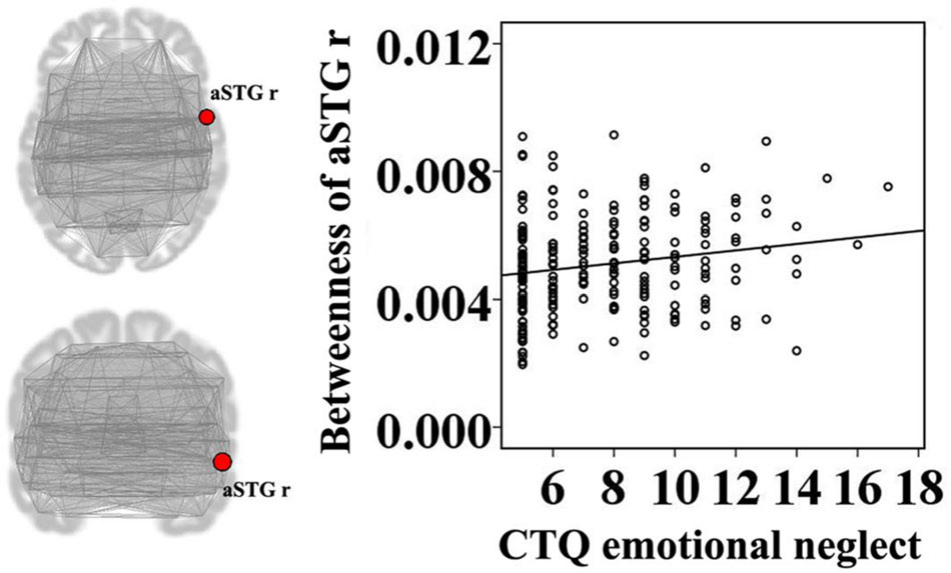
Correlations between the emotional neglect score and value of network property. The scatter diagram showed that betweenness of the aSTG r was positively correlated with the emotional neglect score. aSTG r: right anterior superior temporal gyrus; CTQ: Childhood Trauma Questionnaire.

**FIGURE 3:**
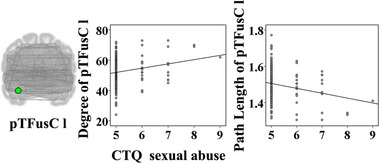
Correlations between the sexual abuse score and values of network properties. The scatter diagrams showed that the sexual abuse score was positively associated with degree of the pTFusC l; path length of the pTFusC l was negatively correlated with the sexual abuse score. CTQ: Childhood Trauma Questionnaire; pTFusC l: left posterior temporal fusiform cortex.

**FIGURE 4:**
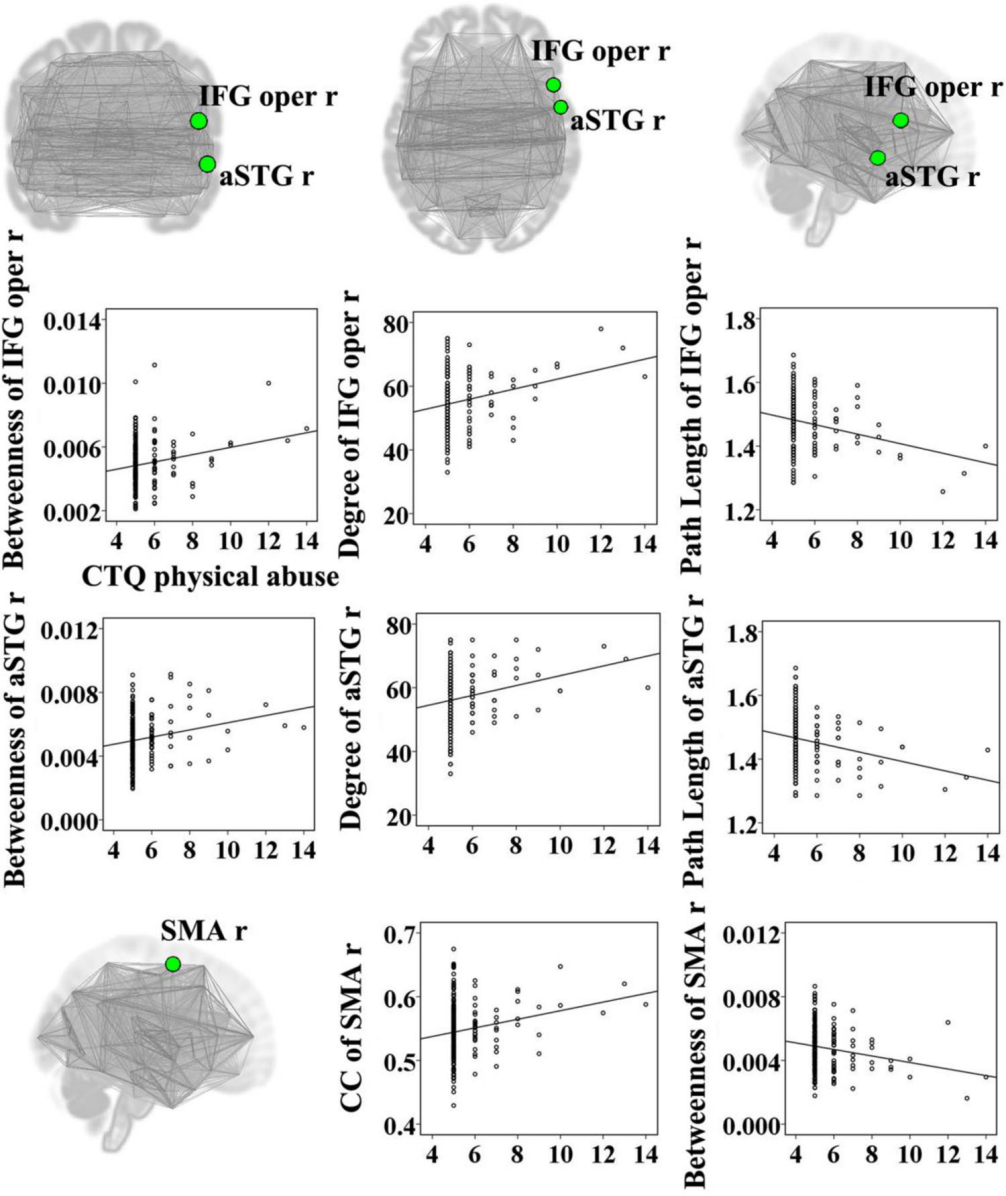
Correlations between the physical abuse score and values of network properties. The scatter diagrams showed that the physical abuse score was positively associated with betweenness and degree of the IFG oper r, as well as the aSTG r; clustering coefficients of the SMA r were positively correlated with the physical abuse score; path length of the IFG oper r, path length of the aSTG r, and betweenness of the SMA r were negatively correlated with the physical abuse score. aSTG r: right anterior superior temporal gyrus; CC: clustering coefficient; CTQ: Childhood Trauma Questionnaire; IFG oper r: right inferior frontal gyrus opercularis; SMA r: right supplementary motor cortex.

**FIGURE 5:**
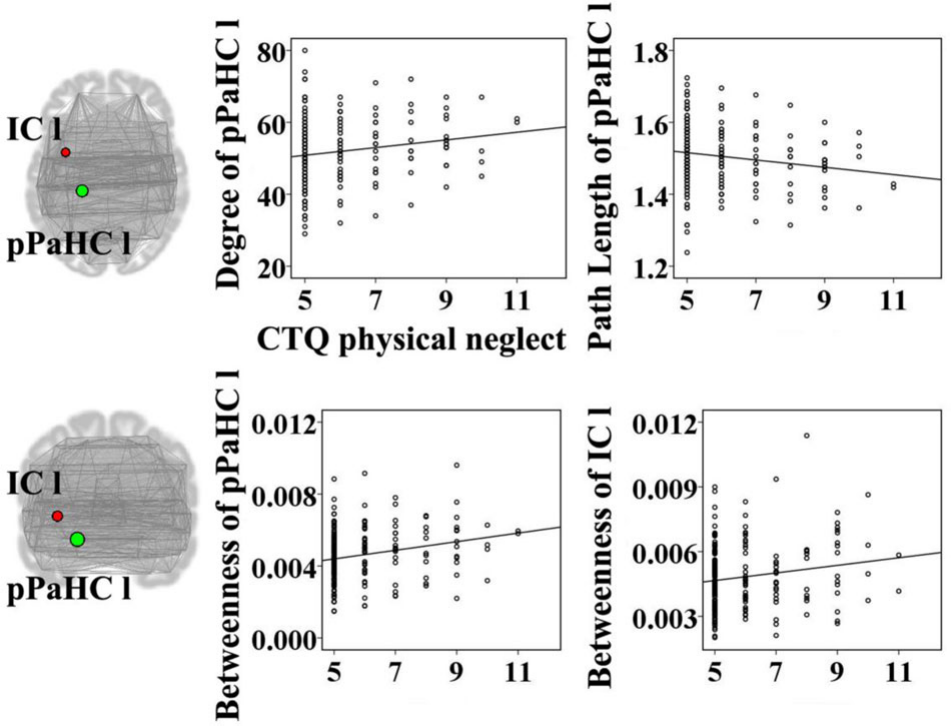
Correlations between the physical neglect score and values of network properties. The scatter diagrams showed that the physical neglect score was positively associated with degree and betweenness of the pPaHC l, as well as betweenness of the IC l; path length of the pPaHC l was negatively correlated with the physical neglect score. CTQ: Childhood Trauma Questionnaire; IC l: left insular cortex; pPaHC l: left posterior division of parahippocampal gyrus.

The emotional abuse score was positively associated with degree of the right lingual gyrus (*t* = 3.07, *p* = .002); path length of the right lingual gyrus (*t* = −3.07, *p* = .002), betweenness of the left posterior middle temporal gyrus (*t* = −2.78, *p* = .006), and betweenness of the left planum temporale (*t* = −2.69, *p* = .008) were negatively correlated with the emotional abuse score (Figure 1). Betweenness of the right anterior superior temporal gyrus (*t* = 2.65, *p* = .009) was positively correlated with the emotional neglect score (Figure 2). The sexual abuse score was positively associated with degree of the left posterior temporal fusiform cortex (*t* = 2.80, *p* = .006); path length of the left posterior temporal fusiform cortex was negatively correlated with the sexual abuse score (*t* = −2.80, *p* = .006) (Figure 3). The physical abuse score was positively associated with betweenness (*t* = 3.04, *p* = .003) and degree (*t* = 3.36, *p* = .001) of the right inferior frontal gyrus opercularis, as well as betweenness (*t* = 2.77, *p* = .006) and degree (*t* = 3.25, *p* = .001) of the right anterior superior temporal gyrus; clustering coefficient of the right supplementary motor cortex was positively correlated with the physical abuse score (*t* = 2.84, *p* = .005); path length of the right inferior frontal gyrus opercularis (*t* = −3.36, *p* = .001), path length of the right anterior superior temporal gyrus (*t* = −3.25, *p* = .001), and betweenness of the right supplementary motor cortex (*t* = −2.64, *p* = .009) were negatively correlated with the physical abuse score (Figure 4). The physical neglect score was positively associated with degree (*t* = 2.72, *p* = .007) and betweenness (*t* = 3.75, *p* < .001) of the left posterior division of parahippocampal gyrus, as well as betweenness of the left insular cortex (*t* = 2.66, *p* = .008); path length of the left posterior division of parahippocampal gyrus (*t* = −2.72, *p* = .007) was negatively correlated with the physical neglect score (Figure 5).

## 4 DISCUSSION

Early trauma in the life of a child can have long‐term effects on his or her mental health later in adulthood. The study showed that both neglect and abuse factors dominantly affect brain several regions involved in cognitive and executive function and information processing such as visual and auditory perception, linguistic comprehension, and movement. In addition, severe abuse even leads to isolated communication between brain regions.

### 4.1 Associations of childhood neglect with disruption of cognitive and executive functions in adulthood

Children neglect is mainly induced by guardian failure to provide basic living and emotional needs when a child grows up and results in the superior temporal gyrus and the development of hallucinations and thought disorder. Structural imaging showed the disorder of superior temporal gyrus, insular cortex, and parahippocampal gyrus may play important roles in the processes. Insular cortex is responsible for executive control function and guidance and maintenance of the body's homeostasis (Bhaya‐Grossman & Chang, 2022), which is probably the embodiment of emotional control function and related to formation of internal receptivity in cognitive process, including perceptual consciousness. The human superior temporal gyrus, including the non‐primary auditory cortex, is the key part of the speech processing. There is a link between pathophysiological changes in the superior temporal gyrus and the development of hallucinations and thought disorder (Steward et al., 2020). Parahippocampal gyrus plays a role in higher cognitive functions, including memory coding and retrieval, and visual space processing, traumatic psychosis altered brain activation, and connection related to memory inhibition (Jaffee, 2017). Our findings of positive association between neglect scores and information transmission efficiency in superior temporal gyrus, insular cortex, and parahippocampal gyrus not only interpret the consequence of childhood neglect on memory and cognition but also further underpin the associations of childhood neglect with altered executive processing.

### 4.2 Associations of childhood abuse with immature ego defense mechanisms and damaged emotion representation

Living environment is the key factor in shaping an individual's emotion comprehension and expression (Livneh & Andermann, 2021). Abused children are frequently exposed to bestial words and negative emotions, caused emotion regulation problems that accompany a child into adulthood. Researchers have proposed multiple potential mechanisms; for example, childhood abuse increases risk for psychopathology. Herein, our results highlighted unique effects of abuse subtypes on brain function and focused on two phenomena: damaged emotion representation and immature ego defense mechanisms (Finzi‐Dottan & Karu, 2006; Yi et al., 2019).

The inferior frontal gyrus opercularis and the superior temporal gyrus are both network hubs in encoding speech features (Brownell et al., 2013; Kitada et al., 2010) had different roles in the identification of emotional facial stimuli (Pollak & Tolley‐Schell, 2003). The increased information transmission efficiency of visual and linguistic cortices that are sensitive to emotional stimuli in our findings means that emotional and physical abuse increase information transmission efficiency in the visual and linguistic cortices, might explain that abused children possessed sensitivity to threatening signals in consisting with a series of studies showing that abused children exhibited preferential attention to angry expressions (Gibb et al., 2009), increased sensitivity in the detection of angry expressions (Liu et al., 2017; Pollak et al., 2009), and have difficulty in transitioning from an angry facial stimulus to other emotions (Curtis & Cicchetti, 2011). The supplementary motor cortex is the center for motor planning, involved with the translation of thought planning into motor programs (Tanaka & Kirino, 2017). Our results found that the reconfiguration of the supplementary motor cortex reflected that maltreating subjects engaged in more defensive behavior validation and fewer prototypical expressions of emotions. The damage of supplementary motor cortex was primarily impaired in the abstract‐emotional word processing (Dreyer et al., 2015). In our results, internally efficiency of the supplementary motor cortex was increased, whereas externally communication was isolated in physical abused subjects. Analogously, betweenness of the posterior middle temporal gyrus and the planum temporale was negatively correlated with the emotional abuse score. Auditory regions, such as middle temporal gyrus and planum temporale, integrate acoustic singles with amygdala input to form emotion‐specific representations (Leitman et al., 2010). In our results, the externally isolated communication in auditory regions impaired emotion representation and increased risk for emotion regulation problems.

Immature defense mechanism was easily shaped in childhood and adolescence in order to cope with inward and outward trauma, regulate subjective experiences of hypochondriasis, fantasy, dissociation, acting out, hostility, and passive aggression (Paris et al., 1996; Vaillant, 1994). The ability of face recognition is critical for normal ego defense mechanism. In accordance to face‐selective signals reliably inducing alteration in the temporal fusiform cortex (McGugin & Gauthier, 2016; McGugin et al., 2016), the sexual abuse score was positively associated with network efficiency of the posterior temporal fusiform cortex in our study also confirms this point.

### 4.3 Limitations

There were several limitations in this study. First, a retrospective self‐report questionnaire of childhood trauma was used. Recalled experience might bias accuracy of trauma experience types. Second, trauma occurrence age and resilience were not included as confounding factors. Whether the span of time experienced in childhood trauma increases the risk for psychopathology in adulthood and whether resilience following childhood maltreatment protects against adult psychopathology are both important and should be analyzed in the future studies. Finally, there is a need for a multilevel perspective to trace the impacts of trauma on pathways from genes to brain to behavior. It would be of great interest to explore biological interactions among brain networks, genes, and behaviors in the future.

## 5 CONCLUSION

Childhood neglect affected subregions in the involvement of cognition and executive function, whereas childhood abuse induced altered brain regions related to ego defense system and emotion representation. Different experiences of childhood trauma biases distinct development of brain function processing and underpin potentially differential risks of specific forms of psychiatric sequelae in adulthood, implicating better intervention strategies for children who exposed to trauma.

## AUTHOR CONTRIBUTIONS

Jianjian Cai and Qin Liu designed the study, analyzed the data, and wrote the manuscript. Jia LI, Sikang Gao, and Dong Liu analyzed the data. Jia LI, Sikang Gao, Yunyun Zhao, and Dong Liu collected data. Qin Liu provided feedback and edited the manuscript. All authors contributed to manuscript revision, read, and approved the submitted version.

## CONFLICT OF INTEREST STATEMENT

The authors declared that the research was conducted in the absence of any commercial or financial relationships that could be construed as a potential conflict of interest.

### PEER REVIEW

The peer review history for this article is available at https://publons.com/publon/10.1002/brb3.2981.

## Data Availability

The raw/processed data required to reproduce these findings cannot be shared at this time as the data also form part of an ongoing study.
